# Novel Homozygous Mutations in the Genes *TGM1*, *SULT2B1*, *SPINK5* and *FLG* in Four Families Underlying Congenital Ichthyosis

**DOI:** 10.3390/genes12030373

**Published:** 2021-03-05

**Authors:** Fozia Fozia, Rubina Nazli, Sher Alam Khan, Ahmed Bari, Abdul Nasir, Riaz Ullah, Hafiz Majid Mahmood, Muhammad Sohaib, Abdulrahman Alobaid, Siddique A. Ansari, Sulman Basit, Saadullah Khan

**Affiliations:** 1 Institute of Basic Medical Sciences (IBMS), Khyber Medical University (KMU), Peshawar 25100, Khyber Pakhtunkhwa, Pakistan; drfoziazeb@yahoo.com; 2 Department of Biotechnology and Genetic Engineering, Kohat University of Science & Technology (KUST), Kohat 26000, Khyber Pakhtunkhwa, Pakistan; sakmarwat79@gmail.com; 3 Department of Pharmaceutical Chemistry, College of Pharmacy, King Saud University, Riyadh 11451, Saudi Arabia; abari@ksu.edu.sa (A.B.); aalobaid1@ksu.edu.sa (A.A.); sansari@ksu.edu.sa (S.A.A.); 4 Department of Molecular Science and Technology, Ajou University, Suwon 16499, Korea; nasirkhan@ajou.ac.kr; 5 Department of Pharmacognosy (MAPPRC), College of Pharmacy, King Saud University, Riyadh 11451, Saudi Arabia; rullah@ksu.edu.sa; 6 Department of Pharmacology, College of Pharmacy, King Saud University, Riyadh 11451, Saudi Arabia; harshad@ksu.edu.sa; 7 Department of Soil Sciences, College of Food and agriculture Sciences, King Saud University, Riyadh 11451, Saudi Arabia; msohaib@ksu.edu.sa; 8 Center for Genetics and Inherited Diseases, Taibah University, Al-Madinah 42353, Saudi Arabia; sbasit.phd@gmail.com

**Keywords:** ichthyosis, whole exome sequencing, splice site variant, *TGM1* and *SPINK5*

## Abstract

Background: Ichthyoses are a large group of hereditary cornification disorders, which are both clinically and etiologically heterogeneous and affect mostly all the skin surface of the patients. Ichthyosis has its origin in an ancient Greek word “ichthys” meaning fish, this is because the ichthyosis patients have dry, thickened, and scaly skin. There is an excess accumulation of epidermal cells resulting in the appearance of continuous and widespread scales on the body. There are many varieties of ichthyosis with a broad spectrum of intensity, severity, and associated symptoms, most of them are extremely rare. Ichthyosis vulgaris is the most frequently occurring type of ichthyoses. Method: The present study consists of four Pakistani ichthyosis families (A, B, C, and D). Whole exome sequencing (WES) approach was used to identify the pathogenic sequence variants in probands. The segregation of these variants in other participants was confirmed by Sanger sequencing. Results: Total four variants including, two splice site (*TGM1*: c.2088 + 1G > A) and (*SPINK5*: c.882 + 1G > T), a missense (*SULT2B1*: c.419C > T; p. Ala140Val), and a nonsense (*FLG*: c.6109C > T; p. Arg2037Ter) variant were identified in families A, C, B, and D, respectively, as causative mutations responsible for ichthyosis in these families. Conclusion: Our study unravels the molecular etiology of the four Pakistani ichthyosis families and validates the involvement of *TGM1, SULT2B1, SPINK5,* and *FLG*, in the etiology of different forms of ichthyosis. In addition, this study also aims to give a detailed clinical report of the studied ichthyosis families.

## 1. Introduction

Congenital ichthyoses are a large group of genetic abnormalities having defective cornification of the skin as a hallmark. The clinical features of all ichthyoses are diverse and highly variable including extensive scaling of the skin, hyperkeratosis associated with variable erythema, xerosis, pruritus, sweating impairment, and suprabasal epidermolysis [1]. Ichthyoses are categorized into nonsyndromic/isolated and syndromic forms, based on their pathophysiology, clinical manifestations, and mode of inheritance. They are inherited in autosomal recessive, autosomal dominant, and X-linked forms [1,2].

To date, five major types of nonsyndromic ichthyoses have been documented, including congenital ichthyosiform erythroderma (OMIM: 242500), ichthyosis vulgaris (OMIM: 146700), lamellar ichthyosis (OMIM: 146750), epidermolytic hyperkeratosis (OMIM: 113800), and X-linked ichthyosis (OMIM: 308100) [1]. In nonsyndromic ichthyoses, the stratum granulosum and stratum corneum are malformed and do not exhibit their normal characteristics, that can be distinguished based on their clinical manifestations, histological findings, structural and biochemical abnormalities of the epidermis as well as genetic etiology [3,4]. Syndromic ichthyoses have also been reported in the literature including, combination with extracutaneous findings such as hair, nails, teeth, bones, exocrine glands, neurologic signs, trichothiodystrophy, and other associated manifestations. Netherton syndrome (OMIM: 256500), Sjogren-Larsson syndrome (OMIM: 270200), Dorfman-Chanarin syndrome (OMIM: 275630), ichthyosis follicularis (OMIM: 308205), and keratitis ichthyosis deafness (KID) syndrome (OMIM: 148210) are some syndromic forms of ichthyoses [5].

Different genes and their sequence variants which are involved and implicated with ichthyoses, disrupt cellular pathways including DNA repair, lipid biosynthesis, adhesion, and desquamation along with many other pathways. Among several pathways involved in pathogenesis, each show features which disturb barrier function [5,6,7,8]. The identification of sequence variants as the cause of genetic diseases has been made very easy and faster by next-generation sequencing (NGS) approaches.

Here we report 10 ichthyosis patients from three consanguineous and a nonconsanguineous Pakistani family, showing the typical phenotypes of ichthyosis including, scaling of the skin, erythema, keratoderma, hyper linearity of the palms and soles, and Pruritus, etc. The observed phenotypes were segregated with two homozygous splice site variants in *TGM1* and SPINK5, a homozygous missense variant in SULT2B1, and a homozygous nonsense variant in FLG, respectively, identified through the whole exome sequencing (WES) approach.

## 2. Materials and Methods

### 2.1. Ethical Approval

Strictly following the recommendations of Helsinki declarations this research study was approved by the Ethical Review Committee (ERC) of Institute of Basic Medical Sciences (IBMS), Khyber Medical University (KMU), Peshawar, Ethical code no Dir/KMU-EB/HM/000741/Dated 8/10/2020 and Ethical and Research Committee of Kohat University of Science and Technology (KUST), Kohat, Khyber Pakhtunkhwa Pakistan, Ref.No.VC-KUST/ethicalcommittee/16-25/26.04.2016. Written consent was taken from all the participants including their parents/legal guardians.

### 2.2. Family Recruitment

**Figure 1:**
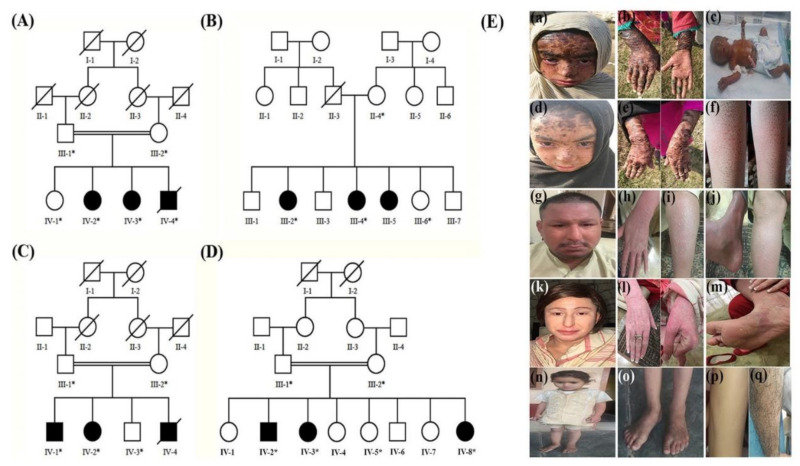
(**A**–**D**) Pedigrees of the families A, B, C, and D segregating different forms of ichthyoses in autosomal recessive manner. Double lines are indicative of consanguineous union. Circles and squares represent females and males, respectively. Clear symbols represent unaffected individuals while filled symbols represent affected individuals. The individual numbers labeled with asterisks indicates the samples which are available for the studies. (**E**) Clinical phenotype of different forms of ichthyosis in the four families. (**a**,**b**,**d**,**e**) Female patients IV-2 and IV-3 from Family A showing classical features of LI and (**c**) newborn IV-4 from the same family with collodion membrane. (**f**) Patient III-2 from Family B with clinical representation of ARCI. (**g**–**m**) A male (IV-1) and a female (IV-2) patient of Family C show the typical clinical phenotypes of NS. (**n**–**q**) Two affected patients IV-2 and IV-8 of Family D with phenotypic features of ichthyosis vulgaris.

We recruited four unrelated Pakhtoon tribe families (A, B, C, and D) from different regions of Khyber Pakhtunkhwa (KP) province of Pakistan (Figure 1). Pedigrees were constructed based on the interview from well informed elder members of the respective families. The autosomal recessive mode of inheritance was assumed as shown by all four pedigrees presentation.

### 2.3. Blood Sample Collection

Venous blood samples were collected from 21 members of four families in total, including three affected (IV-2, IV-3, IV-4) and three phenotypically healthy individuals (III-1, III-2, IV-1) in Family A, two patients (III-2, III-4) and two healthy individuals (II-4, III-6) in Family B, two patients (IV-1, IV-2) and three healthy individuals (III-1, III-2, IV-3) in Family C and three patients (IV-2, IV-3, IV-8) and three healthy participants (III-1, III-2, IV-5) in Family D in EDTA coated tubes (BD Vacutainer K3, Franklin Lakes NJ, USA). A thorough clinical examination of all the affected members of each family was performed by an expert dermatologist (Table 1).

### 2.4. Genomic DNA Extraction

The human genomic DNA was isolated from the peripheral blood of all the participants by using the genomic DNA extraction kit (QIAGEN, Germantown, MD, USA) following the manufacturer’s guidelines.

### 2.5. Whole-Exome Sequencing and Segregation of Rare Variants through Sanger Sequencing

A total of 70 ng/μL genomic DNA of a patient from each family, was used for exome sequencing. For WES enrichment, Nimblegen SeqCap EZ Human Exome Library v2.0 kit was used. Illumina HiSeq 4000 (San Diego, CA, US) was used for running the generated libraries through a paired-end 100-bp protocol [9]. The exome data was analyzed by Cologne Center for Genomics (CCG) Varbank pipeline v2.26 (https://varbank.ccg.uni-koeln.de, accessed on 11 November 2020) (external users). The data mean coverage was fixed as 77%, while 92.6 and 96.6 at 20× and 10×, respectively, were fixed as coverage of the targeted bases. The value of <0.01 was established as the minor allele frequency (MAF value) of the variants by consulting genomAD [10]. A total of 512 in-house database exomes and 67 exomes from the Pakistani population were consulted as controls. The exome data was filtered and *TGM1*, *SULT2B1*, *SPINK5*, and *FLG* rare variants were favored for cosegregation in the remaining participants of the respective families. The online prediction tools including Mutation Taster( NCBI 37/ Ensembl 69, http://www.mutationtaster.org/, accessed on 11 November 2020), PROVEAN v1.1.3, SIFT (https://sift.bii.a-star.edu.sg/SIFT for missense variants 6.2.1, accessed on 11 November 2020), PolyPhen2.0 (http://genetics.bwh.harvard.edu/pph2, PolyPhen v2.0, accessed on 11 November 2020), I-Mutant (http://gpcr2.biocomp.unibo.it/cgi/predictors/I-Mutant3.0/I-Mutant3.0.cgi, accessed on 11 November 2020, VarSome (https://varsome.com/, accessed on 11 November 2020), and CRYP-SKIP (http://cryp-skip.img.cas.cz/, accessed on 11 November 2020) were used to see their pathogenicity and ill effect on the physiology and morphology of their respective proteins/transcripts (Table 2).

*TGM1* (NM_000359.2), *SULT2B1* (NM_177973.1), *SPINK5* (NM_001127698.1), and *FLG* (NM_002016.1) reference sequences were obtained from the University of California Santa Cruz (UCSC) genome database browser (https://genome.ucsc.edu/ accessed on 23 November 2020). Primer3 Plus (http://www.bioinformatics.nl/cgi-bin/primer3plus/primer3plus.cgi/, accessed on 2 December 2020) was used for designing the primers for PCR amplification (Supplementary data, Table S1, Primers used for the amplification of the regions of interest). The amplified PCR products were sequenced by using the BigDye chemistry v3.1 on the ABI3730 genetic analyzer (Applied Biosystems, Foster City, CA, USA). BioEdit version 6.0.7 (http://www.mbio.ncsu.edu/BioEdit/bioedit.html/, accessed on 10 December 2020) was used as sequence alignment tool against the reference sequence.

### 2.6. Homology Modeling

The target sequences of human FLG and *SULT2B1* proteins were obtained from the UniProt database (https://www.uniprot.org/ accessed on 14 December 2020) for homology modeling. To find appropriate structural templates for FLG and SULT2B1, the PSI-BLAST was run against the Protein Data Bank (PBD). PDB ID:1WXR and PDB ID:1Q1Q have been used as templates for the structure of FLG and SULT2B1 proteins and their mutants [11,12] and were modeled via MODELLER [13]. Finally, wild and mutant models were visualized by PyMol v2.4 (https://pymol.org/ accessed on 16 December 2020).

## 3. Results

### 3.1. Clinical Findings

**Family A:** In Family A, two sisters (IV-2 and IV-3) aged 8 and 6 years, respectively, were born to their healthy parents at 40 weeks of gestation, with no history of lamellar ichthyosis in the family. Both of these sisters were born as collodion babies and now the extremities have broad, dark brown, plate-like scales and small adherent facial and trunk scales (Figure 1E(a,b,d,e)). The underlying skin was diffusely erythematous. Other symptoms include hyperlinearity of the palms and soles, diffuse clubbing of fingernails, eclabium, ectropion, allergic rhinoconjunctivitis, normal scalp hairs with whitish thick seborrheic flakes, scanty eyebrows, no eyelashes, and chronic otitis media. Their teeth appeared normal. They were also suffering from pruritus, anhidrosis, heat intolerance, lack of skin elasticity, and recurrent skin infections. The skin infections exacerbate in winter and alleviate in summer. The third affected male infant (IV-4) was also born with the collodion membrane but died after 40 days of delivery due to failure to thrive (Figure 1E(c)). The detailed clinical phenotypes are reported in Table 1.

**Family B:** Both the affected members (III-2) and (III-4) of Family B, presenting an autosomal recessive congenital ichthyosis (ARCI) phenotype, were born to their healthy unrelated parents through normal vaginal delivery at 38 weeks of gestation with no history of ARCI disease in the family. Both exhibited generalized scaling consisting of medium-sized brown to grayish scales on the body. Hyperkeratotic plaques ranged from mild-to-moderate grades around the trunk. A few areas such as the face, ears, and middle part of the palms, soles, axillary region, and the popliteal fossa were normal [Figure 1E(f)]. They also showed keratoderma, eclabium, ectropion, pruritus, anhidrosis, atopic dermatitis, heat intolerance, and decreased elasticity of the skin. The condition improves in hot weather but exacerbates during the cold season Table 1.

**Family C:** The analyzed individuals (IV-1 and IV-2) in this family are affected with Netherton syndrome. One male baby died after 2 weeks of birth before inclusion in the study. The clinical manifestations of the affected individuals were strongly suggestive of NS [Figure 1E(g–m)]. The female patient IV-2 (28 years old), presented mild erythematous scales on her body. The number and severity of lesions had progressively increased in adulthood. Physical examination revealed alopecia, white flake-like lesions on her scalp, eclabium, ectropion, thin eyebrows, absent eyelashes, numerous slightly erythematous grey/white eczematous-like localized patchy skin lesions and desquamative plaques, absent body hair, hypohidrosis, diffuse palmoplantar keratoderma, and heat intolerance [Figure 1E(k–m)]. The patient also suffered from pruritus, severe scratching lesions, often causing restlessness and sleeplessness, atopic dermatitis, allergic rhinitis, and recurrent skin and systemic infections which especially flared up in winter. The other affected member IV-1 (31 years old male) has similar clinical features but with less severity as compared to those of her sister [Figure 1E(g–j)]. Both the patients displayed normal intellect with no evidence of any other systemic manifestations (Table 1).

**Family D:** The affected females (IV-3 and IV-8) and the affected male (IV-2) of Family D displaying ichthyosis vulgaris phenotypes, were born to healthy consanguineous parents with congenital erythroderma, mild peeling and dryness of the skin with moderate severity especially, in the groin region and lower legs. On examination, both the members showed generalized mild, dry and whitish to light brown scaly flakes on the skin, prominent on the extensor surfaces of limbs, lower abdomen, foot and soles. Whitish seborrheic plaques were also visible on the extremities. The affected individuals also showed erythema of skin, keratoderma, pruritus, atopic dermatitis, allergic rhinitis, allergic rhinoconjunctivitis, hypohidrosis, heat intolerance, and recurrent infections [Figure 1E(n–q)]. See (Table 1).

### 3.2. Screening of Pathogenic Sequence Variants

By analyzing the WES data, we identified rare homozygous sequence variants in four genes: *TGM1* (OMIM: 190195; 14q12, c.2088 + 1G > A), *SULT2B1* (OMIM: 604125; 19q13.33, c.419C > T; p. Ala140Val), *SPINK5* (OMIM: 605010; 5q32, c.882 + 1G > T), and *FLG* (OMIM: 135940, 1q21.3, c.6109C > T; p. Arg2037Ter) in affected members of Families A, B, C, and D, respectively. The affected individuals were homozygous for the identified pathogenic sequence variants while, the obligate carriers were heterozygous. To exclude the possibility of polymorphism for novel mutations, 100 unrelated ethnically matched healthy controls were screened, which eliminated the risk of these novel mutations being nonpathogenic polymorphisms.

**Figure 2:**
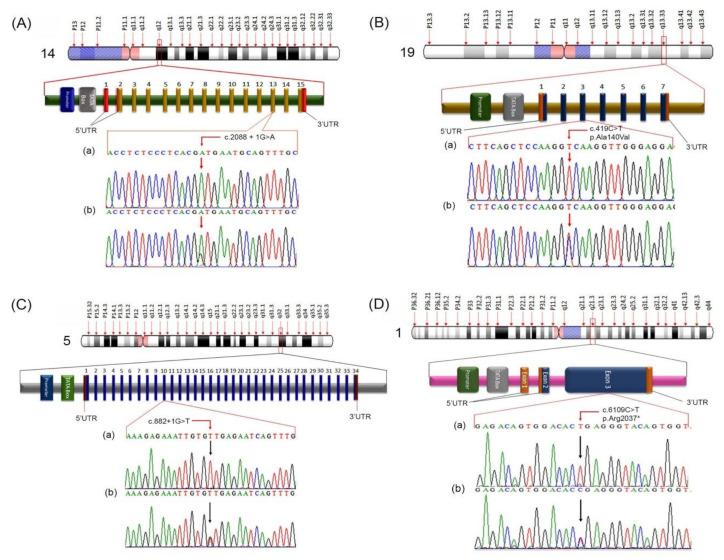
Chromosomal location, hypothetical structure and sequence chromatogram of *TGM1*, *SULT2B1*, *SPINK5*, and *FLG* variants, showing the positions of genetic alterations in the in the present study. (**A**) Chromosomal location and hypothetical structure of *TGM1* containing all 15 exons along with sequence analysis of *TGM1* variant. (**a**) shows a partial sequence of exon-13 of *TGM1* in an affected member (IV-2) with novel splice site mutation involving a homozygous G→A transition in the splice donor site of intron 13 (c.2088 + 1G > A) and (**b**) shows sequence of an unaffected heterozygous carrier (III-2) in Family A. (**B**) Panel B presents chromosomal location and hypothetical structure of *SULT2B1* gene with its six exons. (**a**) shows partial sequence of exon-3 of *SULT2B1* in an affected (III-2) and (**b**) in an unaffected member (II-4) of Family B presenting a novel homozygous missense variant (c.419C > T; p. Ala140Val), where the homozygous deletion of C nucleotide and the simultaneous insertion of T nucleotides is evident in the affected member sequence. (**C**) shows chromosomal location and hypothetical structure of *SPINK5* gene along with its 34 exons. (**a**) It shows a partial DNA sequence of exon-10 of *SPINK5* representing a novel homozygous splice site variant (c.882 + 1G > T) in an affected member (IV-1) and (**b**) in a heterozygous carrier (III-1) of Family C. Panel (**D**) is showing Chromosomal location and hypothetical structure of *FLG gene* containing three exons, along with sequence analysis of *FLG* variant. (**a**) It shows a partial sequence of exon-3 of *FLG* in an affected member (IV-2) with homozygous nonsense variant in the *FLG* (c.6109C > T; p. Arg2037Ter) and (**b**) sequence of an unaffected heterozygous carrier (III-1) in Family D.

In Family A, a novel homozygous splice site mutation in the splice donor site of intron 13 (c.2088 + 1G > A) in *TGM1* was identified. The segregation study revealed that the variant was inherited from both the normal heterozygous parents (Figure 2A). It results in the disruption of the splice donor site of *TGM1* and a set of 54 nucleotides (+8454 to +8509) are deleted, which in turn leads to an in-frame pathogenic deletion of 18 amino acids ranging from 679 to 696. This variant in *TGM1* has neither been documented in gnomAD [10] nor in HGMD [14], in Clinvar [15] and Pakistan Genetic Mutation Database (PGMD) [16], thus is predicted to be novel.

**Figure 3:**
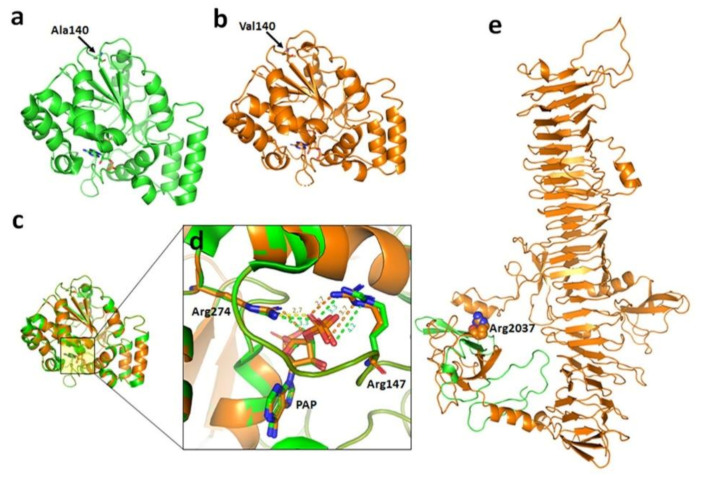
(**a**) Homology model of wild and (**b**) mutant type of SULT2B1 protein. Residues at position 140 are shown in stick model. (**c**) The superimposed structure of wild (green color) and mutant (yellow color) SULT2B1. (**d**) The zoom-up view of PAP binding site in wild type and mutant display difference sin distance which suggest the p. Ala140Val may contribute to the cofactor binding. (**e**) Homology model of FLG from residues 1960–2854. The residue Arg2037 is shown with sphere representation. The orange color ribbon is absent in the p. Arg2037Ter (mutant form).

In Family B, a novel homozygous missense mutation (c.419C > T; p. Ala140Val) in SULT2B1 was identified (Figure 2B). Here, the Pro140 residue at the loop region does not directly bind Adenosine-3’-5’-diphosphate in the crystal structure described by Lee et al. (2003) [12]. However, the comparative study of the wild and mutant protein (p. Ala140Val) displays a difference in distance between the PAP and active site residues. The new Val140 residue very likely disrupts this binding by displacing Arg147 and Arg274, strengthening the norm that missense mutations quite possibly cause the disease by disrupting the association between SULT2B1 and its cofactor “PAP” (Figure 3a–d).

In Family C, a homozygous splice site variant (c.882 + 1G > T) in *SPINK5,* was identified, causing Netherton Syndrome (Figure 2C). The pathogenicity of this variant was confirmed by in silico online prediction tools (VarSome and CRYP-SKIP) suggesting that it may mediate the complete loss of 5’ donor site and may be involved in the exon skipping.

To the best of our knowledge, *SULT2B1* and *SPINK5* variants identified in families B and C (Figure 2B,C), respectively, in this study, have never been documented in homozygous state in ichthyosis families both globally and in the South Asian subcontinent including Pakistani population before [16].

In Family D, a nonsense variant (c.6109C > T; p. Arg2037Ter) in exon 3 of FLG was identified and its segregation was confirmed in other participants by Sanger sequencing (Figure 2D). A premature stop codon is induced in the transcript of the mutant at position 2037, hence, the last 2024 amino acids are not produced and translation stops at 2037 position, leading to the loss of normal protein function (Figure 3e). The Arg2037Ter variant has not been reported earlier in the homozygous state in the South Asian subcontinent including Pakistan.

## 4. Discussion

In this study, we recruited a total of 10 ichthyosis patients from four unrelated Pakhtoon families from different regions of Khyber Pakhtunkhwa (KP), Pakistan. Detailed clinical and physical examination revealed abnormal phenotypes such as abnormal scaling and erythema of the skin, keratoderma and hyperlinearity of the palms and soles, alopecia, pruritus and atopic dermatitis, etc., caused by pathogenic variants in four different genes (*TGM*, *SULT2B1*, *SPINK5*, and FLG) identified by WES. Previous studies have shown that all these four genes are involved in causing different types of ichthyosis [17,18,19,20].

Here, we present a comprehensive report of clinical and molecular characterization of ichthyosis Family A of Pakistani origin, showing the clinical hallmarks of a lamellar form of autosomal recessive congenital ichthyosis. The affected members showed typical phenotypes including the presence of collodion membrane during birth, along with large and thick, dark-brown plate-like adherent scales on the face, ears and extremities. The *TGM1* is located on chromosome 14q12, and comprises 14,095 bp in size (2454 bp coding sequence), and houses 15 exons (GenBank NM_000359.2). The protein (90 kDa) encoded by *TGM* has many functional domains including Transglut N (117–235 aa), Transglutaminase/protease-like homologs domain (TGc) (348–468 aa), and Transglut C domain (587–691 aa and 699–796 aa) [21]. The variant identified in our patients is positioned in the Transglut C domain that speculatively leads to protein aberrations and affects the enzymatic activity, explaining a dramatic development of postnatal epidermal cornification. The *TGM1* functionally provides instructions for making a transglutaminase 1 (TGase1) enzyme. TGase1 is among the eight catalytic transglutaminases recognized in humans and also among three transglutaminases situated in the epidermis [22]. TGase-1, a Ca2+-dependent, membrane-bound enzyme, is involved in the formation of the cornified cell envelope(epidermis) by cross-linking soluble cytoplasmic proteins onto the plasma membrane that provides strength and stability to the epidermis layer [23]. A x-hydroxy-ceramide is then covalently bound to CCE, a process that also involves TGm-1 [24]. TGase-1 is responsible for catalyzing the critical Ne-(c-glutamyl) lysine crosslinking of preserver proteins such as involucrin and loricrin and attachment of long chain omega-hydroxyceramides to involucrin by ester bond during the formation of the CCE. The CCE acts as a mechanical barrier and protects against water loss and infectious agents [25]. *TGM1* mutations are the predominant and most convincing causes of autosomal recessive congenital ichthyosis (ARCI; OMIM: 190195, 242100, 242300), a rare, nonsyndromic and heterogeneous disorder of cornification, having three distinct clinical subtypes, including, lamellar ichthyosis (LI; OMIM: 242300), harlequin ichthyosis (HI; OMIM: 242500) and congenital ichthyosiform erythroderma (CIE; OMIM: 242100) [26]. Based on the previous studies, *TGM1* has been one of the most common candidates responsible for LI [21,26,27,28,29]. Knockout studies on *TGM1* in mice have shown erythematous and translucent tight skin, a prominent sign of a collodion membrane [28,29,30]. Neonatal TGM1−/− knockout mice have displayed severe feeding difficulties and consequently died within 4–5 h of birth because of severe dehydration [30].

The genetic analysis of Family B showed a novel homozygous missense variant (c.419C > T; p. Ala140Val) in *SULT2B1*, revealing its lethal role in the causation of ARCI. The variant present in our patients is located in the sulfotransferase domain that may affect the protein physiology and stability. The *SULT2B1* (OMIM: 604125) is located on 19q13.33 and has seven coding exons. Previous literature and studies on the model organisms have shown that genetic alterations in the *SULT2B1* have been strongly associated with ARCI [31,32,33]. Heinz et al. (2017) reported four mutations including nonsense, missense, and splice-site in three unrelated ichthyosis families using WES and multigene panel screening approaches [31]. Another group of researchers reported a cohort of 125 ARCI consanguineous families for screening the genetic alterations. They used a targeted NGS panel comprising 38 ichthyosis related genes and found pathogenic variants in the *SULT2B1* [26]. *SULT2B1* provides cholesterol sulfate, an active regulator of the keratinocyte differentiation, and thus has a pivotal role in homeostasis and barrier function of the human skin.

Affected individuals in Family C showed the main phenotypes of Netherton syndrome (NS; OMIM: 256,500), including congenital ichthyosiform erythroderma, persistent atopic manifestations and high levels of serum immunoglobulin E (IgE) in the blood. The disease is very severe, painful and fatal in the case of infants and newborns [34]. A novel homozygous splice site variant (c.882 + 1G > T) in *SPINK5* was identified in this family, which in its mutated form causes the NS phenotypes. In understanding the pathogenesis of the disease, Spink5 knock-out murine models have been important and have driven NS patient investigations. They have recognized kallikreins as the main LEKTI targets. In particular, the unopposed operation of kallikrein-related peptidase 5 (KLK5) plays a crucial role in initiating a dual biological cascade that has major impacts on the integrity and permeability of the skin barrier and also triggers skin inflammation and allergy through PAR-2-dependent and PAR-2-independent pathways [18,35]. *SPINK5* is located on 5q32 and has 34 coding exons. The *SPINK5* variant found in our patients exhibited a very close clinical match with those of the patients described in the previous literature [36,37,38].

Molecular analysis of Family D revealed a novel homozygous nonsense variant (c.6109C > T; p. Arg2037Ter) in three affected siblings in *FLG*, causing ichthyosis vulgaris phenotypes in its mutated form. The effect of the induced stop codon at amino acid position 2037 would cause the protein truncation and likely be targeted by nonsense-mediated mRNA decay (NMD) [39].

*FLG* has been found in association with different clinical manifestations such as asthma, eczema, atopic dermatitis, and ichthyosis vulgaris. *FLG* is located on 1q21.3, consisting of three coding exons. *FLG* is involved in several metabolic processes, e.g., acts as a hub in driving an intricate chain of interrelated functions, in the development of structural and chemical barriers, skin hydration, and regulation of epidermal homeostasis [40]. In the mammalian epidermis, the FLG protein plays a chief role in the aggregation of keratin intermediate filaments. Firstly, it is produced in the form of a precursor polyprotein molecule in the keratohyalin granules and then is proteolytically processed into the filaggrin molecules [40,41]. Further studies on knockout models have described that FLG protein is crucial for the development of the skin barrier and genetic alterations in the *FLG* lead to major predisposing factors for atopic disorders [42].

## 5. Conclusions

Taken together, the present study of four Pakistani families supports previous research that biallelic mutations in *TGM1*, SULT2B1, SPINK5, and *FLG* cause human ichthyoses. Our findings expand the mutation spectrum and add to the knowledge on clinical heterogeneity associated with *TGM1*, SULT2B1, SPINK5, and *FLG* variants, it also helps in genetic counseling and accurate prenatal diagnosis for parents of at-risk individuals to facilitate decision making about the continuation of the next upcoming pregnancy [43,44], especially in a region like Pakistan, where the custom of consanguineous marriages prevails at a much higher rate.

Prenatal diagnosis is usually done by extracting DNA from a sample of chorionic villus (CVS) obtained at 10 or 13 weeks of gestation or by extracting DNA from fetal cells obtained by amniocentesis at approximately 15–18 weeks of gestation. For families in which disease-causing mutations have been reported previously, preimplantation genetic diagnosis (PGD) may be applicable. Using in vitro systems and animal models, future clinical trials and laboratory studies will enable us to elucidate the pathogenicity sequence of the disease that will contribute to a broader understanding of the genetic, molecular, and pathophysiological aspects of ichthyoses and will lead to the discovery of novel treatments.

## Figures and Tables

**Table 1:** Clinical phenotypes of the patients in four Pakistani ichthyosis families.

Phenotype/Features	Family A	Family B	Family C	Family D
Type of ichthyosis	Severe lamellar ichthyosis	Congenital ichthyosis (AR)	Netherton syndrome	Ichthyosis vulgaris
Ethnicity	Pakistani	Pakistani	Pakistani	Pakistani
Consanguinity	+	-	+	+
Pregnancy	Full term/uneventful	Full term/uneventful	Full term/uneventful	Full term/uneventful
Reduced fetal movement	−	–	–	–
Delivery by CS	–	–	–	–
Skin condition at birth	Collodion membrane at birth	Collodion membrane at birth	Presented with congenital erythroderma and scaling of moderate severity	Presented with congenital erythroderma and dryness of skin of moderate severity especially in the groin region and lower legs
Skin involvement	Large, thick, dark brown, plate-like adherent scales on forehead, face, ears, the extremities, and trunk. The underlying skin is diffusely erythematous	Generalized scaling consisting of medium sized brownish to gray scales on the body. Hyperkeratotic plaques over the trunk varied from mild to moderate grade. A few areas such as the face, ears, and the middle part of the palms and soles, axillary region and the popliteal fossa are not affected	Skin presents with diffuse superficial whitish scaling, eczematous-like localized patchy lesions and minute nodular papules on the body. Patchy erythroderma of the skin also visible	Generalized mild dry and whitish to light brown color scaly flakes on the skin prominent on the extensor surfaces of limbs, lower abdomen and on the foot and soles, whitish seborrheic plagues are also visible on the extremities
Erythema of skin	+	+	+	+
Keratoderma	+	+	+	+
Hyperlinearity of the palms and soles	+	+	+	+
Hair loss	Normal scalp hairs with whitish thick seborrheic flakes. Scanty eyebrows and no eye lashes	­–	Alopecia with sparse eyebrows and no eye lashes	­–
Nail changes	Diffuse clubbing	­–	­–	­–
Onychauxis	+	­–	­–	­–
Teeth abnormalities	­–	­–	­–	­–
Ear abnormalities	­–	­–	­–	­–
Hand abnormalities	­–			
Eclabium	+	+	­–	­–
Ectropion	+	+	­–	­–
Pruritus	+	+	Severe with scratching lesions, often causing restlessness and sleeplessness	+
Urticaria	­–	­–	+	+
Anhidrosis	+	+	­–	­–
Atopic dermatitis	+	+	+	+
Allergic rhinitis	­–	­–	+	+
Hypohydrosis	­–	­–	+	+
Asthma	­–	­–	+	+
Allergic rhinoconjunctivitis	+	+	+	+
Heat intolerance	+	+	+	+
Hearing loss	­–	­–	+	­–
Choronic Otitis Media	+		+ ­	­–
Joint movement limitations	+	­–	­–	­–
Lack of skin elasticity	+	+	+	+
Seasonal relation	Condition aggravates in winter	Condition aggravates in winter	Condition aggravates in winter	Condition aggravates in winter
Recurrent skin infections	+	+	+	+
Intellectual disability	­–	­–	­–	­–
Respiratory system abnormalities	­–	­–	­–	­–
Renal system abnormalities	­–	­–	­–	­–
Heart abnormalities	­–	­–	­–	­–

**Table 2:** Bioinformatics analysis of the identified sequence variants in four Pakistani ichthyosis families.

S. No.	Gene	OMIM Number	cDNA Change	Amino Acid Change	Mutation Taster	PROVEAN	SIFT	PolyPhen2.0	I-Mutant	VarSome	CRYP-SKIP
1.	*TGM1*	190195	c.2088 + 1G > A	NA	Diseasing causing Prob: 1	NA	NA	NA	NA	Pathogenic	P_CRE_ = 0.31
2.	*SULT2B1*	604125	c.419C > T	p. Ala140Val	Disease causing Prob: 0.99	Deleterious score: −3.726	Tolerated	Probably damaging score: 1.000	Protein stability decreases	NA	NA
3.	*SPINK5*	605010	c.882 + 1G > T	NA	Disease causing Prob: 0.99	NA	NA	NA	NA	Pathogenic	P_CRE_ = 0.06
4.	*FLG*	135940	c.6109C > T	p. Arg2037 *	Disease causing Prob: 1	NA	NA	NA	NA	NA	NA

Abbreviations; NA: not applicable, P_CRE_: probability of cryptic splice site activation; OMIM: Online Mendelian Inheritance in Man.

## Data Availability

The data presented in this study are available on request from the corresponding author. The data are not publicly available due to privacy and ethical issues.

## Supplementary Materials

The following are available online at https://www.mdpi.com/2073-4425/12/3/373/s1, Table S1: Primers used for the amplification of the regions of interest.
