# Supplementary material for: Novel Homozygous Mutations in the Genes TGM1, SULT2B1, SPINK5 and FLG in Four Families Underlying Congenital Ichthyosis
Source: Genes (Basel). 2021 Mar 5;12(3):373. doi: 10.3390/genes12030373 (PMC7999895; doi:10.3390/genes12030373)
Supplement: Supplementary file 1 [file genes-12-00373-s001.pdf]

Table S1. Primers used for the amplification of the regions of interest

S. No.	Primer	Sequence	Product	Tm
1.	TGM1-F	CCTGTAAGTGCTCCTTACCC	777bp	56.4°C
	TGM1-R	GGAAGACGACATTGGTGAG		56.5 °C
2.	SULT2B1-F	GCTCGATTTCTCCCAACAG	363bp	58.4 °C
	SULT2B1-R	GCTAGATCAGAGTCAGGCACA		58.2 °C
3.	SPINK5-F	TTTGGGAACTGGAATGTCTT	576bp	57.1 °C
	SPINK5-R	TTGCCTCCATATACAGCTAGAA		56.8 °C
4.	FLG-F	AGTCTTCCTCTCGTGGACA	416bp	55.7 °C
	FLG-R	CTGATCATAATGGGATCCTTG		57.0 °C
